# Atlas and Epitome of Operative Surgery

**Published:** 1899-12

**Authors:** 


**Atlas and Epitome of Operative Surgery.** By Dr. OTTO ZUCKERKANDL. Edited by J. CHALMERS DACOSTA, M.D. Pp. 395. London: The Rebman Publishing Co. (Ltd.) Philadelphia: W. B. Saunders. 1898.

Intended as an elementary guide for students, this work, though written in a clear style and in most respects embodying the present knowledge of its subject, would have been more valuable if the description of some of the operations had been less scanty: its pages abound with illustrations, of which the coloured plates are very clear and well executed; some of the woodcuts however might, in a later edition, be improved or omitted. Some English names appear in the text, but our vanity is a little upset by the omission of many which we think are of world renown.

We do not often now use the old sliding artery forceps as mentioned under amputations; their place has been taken by the more convenient catch forceps, which are applied to the vessel much more easily: the buried suture ought to have been noticed in this chapter. We are so imbued with the usefulness of the aneurysm needle in ligation of vessels, that we think it might be mentioned in describing these operations. In performing the operation of temporary division of the lower jaw, it would be well to impress on the student the importance of drilling the holes for the metallic sutures before the jaw is sawn through. The mode of introducing the sutures for holding each half of a Murphy button in place in the divided intestine is certainly not the best; the sutures ought to be shown as going over the raw edge of the bowel. We should like to see in the pages devoted to catheterisation some instructions concerning the disinfection of the surroundings of the meatus, and also of the catheter, as want of this attention leads sometimes to disaster. The portion of the work devoted to plastic surgery is excellent, and the illustrations here are all that could be desired.

